# Preparation of Nitrogen-doped Holey Multilayer Graphene Using High-Energy Ball Milling of Graphite in Presence of Melamine

**DOI:** 10.3390/ma16010219

**Published:** 2022-12-26

**Authors:** Ali Hendaoui, Abdullah Alshammari

**Affiliations:** 1 Physics Department, College of Science and General Studies, Alfaisal University, P.O. Box 50927, Riyadh 11533, Saudi Arabia; 2 National Center for Desalination & Water Treatment Technology, King Abdulaziz City for Science and Technology, King Abdullah Rd, Al Raed, Riyadh 12354, Saudi Arabia

**Keywords:** graphite, melamine, multilayer holey graphene, nitrogen-doping, high-energy ball milling

## Abstract

Holey graphene, consisting of graphene sheets with in-plane nanopores, has recently attracted more attention as it expands graphene applications to other fields inaccessible by the pristine graphene. To ensure an effective implementation of holey graphene in the market, it is crucial to explore new preparation methods that are simple, cost effective, eco-friendly, versatile, and scalable. While ball milling of graphite in presence of exfoliating agents was found very effective in the preparation of graphene (doped and undoped) and graphene-composites, this technique remains unexplored for the preparation of holey graphene. In the present work, Nitrogen-doped multilayer holey graphene sheets were prepared by an all-solid, one-step procedure based on high-energy ball milling of graphite as the starting material in presence of melamine in a shaker-type mill for 1 hour under ambient conditions. Melamine acted simultaneously as an exfoliating agent to enhance the exfoliation of graphene layers and a diluent to protect graphite against the continuous fragmentation into amorphous carbon during the high-energy “shock” mode of ball milling. The high-energy “shock” mode of ball milling of graphite in presence of melamine induced the formation of multilayer defective graphene as an intermediate product before being converted into N-doped multilayer holey graphene after the removal of the in-plane defects during the milling process. The characterization of the final product confirmed the formation of N-doped multilayer holey graphene with a content in nitrogen as high as 12.96 at.%, making it promising for energy storage and energy conversion applications.

## 1. Introduction

Graphene displays a combination of structural, electrical, thermal, and mechanical properties that position it as an important 2-D material to manufacture a wide range of uniquely enhanced products for various sectors, including clean energy, wearable technology, biomedical devices and aerospace [1,2,3].

More recently, porous (i.e., holey) graphene, consisting of graphene sheets with in-plane nanopores, has attracted more attention because it expands graphene applications to other fields inaccessible by the pristine graphene. The pores in the holey graphene induce more reactivity suitable for applications in water treatment, energy storage and energy conversion [4,5,6]. Such features sparked interest in developing new methods to prepare holey graphene. In fact, several methods for the preparation holey graphene have been reported in the literature [4,5,6]. In general, these techniques use graphene, graphene oxide or reduced graphene oxide as a starting material, then induce perforation through different chemical means at high temperatures. Such methods suffer several issues for scale-up, including the high cost of the precursors, the complexity due to a cascade of steps, the use of high temperatures and, usually, the need of a controlled atmosphere. Other environment-related drawbacks such as handling hazardous chemicals could be also considered as a limiting factor for the proposed methods. It is worth mentioning that attempts to develop “green” approaches for the synthesis of holey graphene could also be found in the literature [4]. While such approaches are based on using biomass as the raw material, they still require processing samples at high temperatures over long periods under a controlled atmosphere and, very often, the use of hazardous chemicals. Therefore, there is still a need for the development of simple, cost-effective, eco-friendly, versatile and scalable methods for the preparation of holey graphene.

Ball milling has emerged as an efficient, eco-friendly, cost-effective and scalable approach for the production of graphene, doped graphene and graphene-based composites [7,8,9,10,11,12,13]. This method, which is a top-down method of nanomaterials synthesis, is a process in which graphite is milled either under a controlled atmosphere and/or in presence of organic compounds to induce exfoliation and, ultimately, doping of the graphene sheets. The related process is also referred to as mechanochemical synthesis. For example, a recent report from Navik et al. described a “scalable” production method of graphene by ball milling graphite in supercritical CO_2_ [8]. The proposed synthesis scheme involved a collaborative effect between the high shear forces developed in the planetary ball mill and the intercalation of CO_2_ in the graphite crevices. Another publication from the same group reported an “industrially scalable” method to produce graphene involving the pretreatment of graphite by ball milling in supercritical CO_2_ [9]. N-doped graphene with good electrochemical properties was prepared by milling graphite under nitrogen containing gases [10] of and/or in the presence of nitrogen-rich compounds [11]. The latter is perceived as more cost-effective since it would involve less-expensive equipment as compared to controlled-atmosphere milling approach. Flame-retardant Sn-doped graphene was also prepared by wet ball milling method using expandable graphite and Sn powder as starting materials [12]. Graphene composites, such as SiO_x_/TiO_2_@multilayer-graphene and amorphous MnO_x_@few-layered graphene, were also fabricated by ball milling [13]. The process of “graphene compositing” capitalized on the shear forces during the milling process to exfoliate graphene sheet, and on the heat produced during the milling process to decrease the activation barrier of the exfoliation and to facilitate the anchoring of active materials [13]. 

The ball milling procedures discussed in the literature for the production of graphene (both doped and undoped) and graphene-based composites are based on the low-energy approach of ball milling (i.e., shear exfoliation mode) involving the use of “planetary” ball mills. A typical process in the shear-exfoliation mode involves milling balls that are rolling on the inner surface of the milling vials. The shear forces developed due to the rotation of the milling balls unwrap graphene sheets by breaking the out-of-plane C=C bonds, resulting in the transformation of graphite into graphene. Such a mode, although found effective in terms of yield in graphene, involves milling times that are relatively long, which would add extra cost for the scale-up. Another mode of ball milling, typical of shaker mills, is the high-energy approach (i.e., shock mode) involving the application of vertical forces due to the repeated impacts of the milling ball on the graphite grains, resulting in more fragmentation of the particles that could ultimately lead to amorphous carbon. The shear-exfoliation mode of ball milling is regarded as more effective for graphene exfoliation as compared to its the shock-fracture counterpart. The latter is considered as an unwanted mode of ball milling as it induces the fragmentation of graphite into amorphous carbon rather than producing graphene [14]. This left the high-energy shock mode of milling relatively unexplored in the context of the preparation of graphene and its derivatives. Finally, it is worth mentioning that no reference could be found in the literature about the production of holey graphene via ball milling process, neither in the shear mode, nor in the shock mode. 

Herein, we report on a simple method for the preparation of nitrogen-doped holey multilayer graphene by co-milling graphite and melamine via high-energy ball mill using a shaker mill. More specifically, we demonstrate that the fragmentation process during the high-energy ball milling could be controlled in such a way to produce the perforation of graphene rather than its complete fragmentation into amorphous carbon. In addition to being cost effective, eco-friendly, and versatile, the proposed method is all-solid and is held under ambient conditions (room temperature, atmospheric pressure, air), making it simple and very promising for scale-up.

## 2. Materials and Methods

### 2.1. Sample Preparation

Graphite powder (Merck 99.5%, Merck KGaA, Darmstadt, Germany) and melamine powder (Merck 99%, Merck KGaA, Darmstadt, Germany) were used as the starting materials. In a typical experiment, 25 mg of graphite was milled in presence of 75 mg of melamine (weight ratio of 1:3) in a 5 mL stainless-steel vial (accessory 3127, 19.1 mm diameter × 47.6 mm long) with a stainless-steel ball (6.35 mm diameter) under ambient conditions (room temperature, atmospheric pressure, air) using a SPEX 8000M high-energy shaker mill (Spex® SamplePrep, Metuchen, NJ, USA) operated at 1060 cycles per minute (cpm) for one hour. Because of the amplitude and the frequency of the back-and-forth vial vibration, the normal force of the ball’s impact is important. This confers the high-energy character to this type of mill.

The purpose of using melamine is to facilitate the exfoliation of the graphene sheets. The mechanism through which melamine is thought to exfoliate graphene sheets the ball milling process was investigated both experimentally and theoretically by Leon et al. [15]. In summary, their findings suggest that the exfoliation of the graphene sheets is mediated by the adsorbed melamine molecules on the graphene sheets and formation of 2-D hydrogen-bond networks. Another benefit of using melamine during the milling process is to provide stable covering that adsorbs part of the impact forces to protect graphite from the continuous fragmentation into amorphous carbon. A similar approach, described as diluent-protected milling, was investigated by Buzaglo et al. [16] using different additives added to graphite flakes to minimize the formation of amorphous carbon during the preparation of graphene by dry milling.

### 2.2. Characterization

Scanning electron microscopy images were taken using a JSM-7100F scanning electron microscope (JEOL LTD, Akishima, Tokyo, Japan). X-ray diffraction pattens were obtained using a Rigaku minifle × 600 diffractometer (Rigaku, Tokyo, Japan). UV-Vis absorption measurements on powders’ suspensions in DMF were performed using a Perkin Elmer Lambda 365 spectrophotometer (PerkinElmer Inc., Waltham, MA, USA). 

After washing both pristine (i.e., unmilled) mixture and ball milled powders thoroughly with hot water, Raman spectra was collected using a Thermo Scientific DXR Raman microscope (Thermo Fisher Scientific, Madison, WI, USA). The average in-plane size $L_{a}$ was estimated from Raman spectra using the equation [17]:

(1) $$L_{a}=\left(2.4\times 10^{-10}\right)\lambda_{laser}^{4}{\left(\frac{I_{D}}{I_{G}}\right)}^{-1}$$

where $I_{D}$ and $I_{G}$ are, respectively, the integrated intensities of D and G peaks in the Raman spectra. 

X-ray photoelectron spectroscopy (XPS) spectra were collected using PHI 5000 VersaProbe (ULVAC Physical Electronics, Chanhassen, MN, USA). Transmission Electron Microscopy images were obtained using JEOL JEM-1011 Transmission Electron Microscope (JEOL LTD, Akishima, Tokyo, Japan).

## 3. Results and Discussion

**Figure 1:**
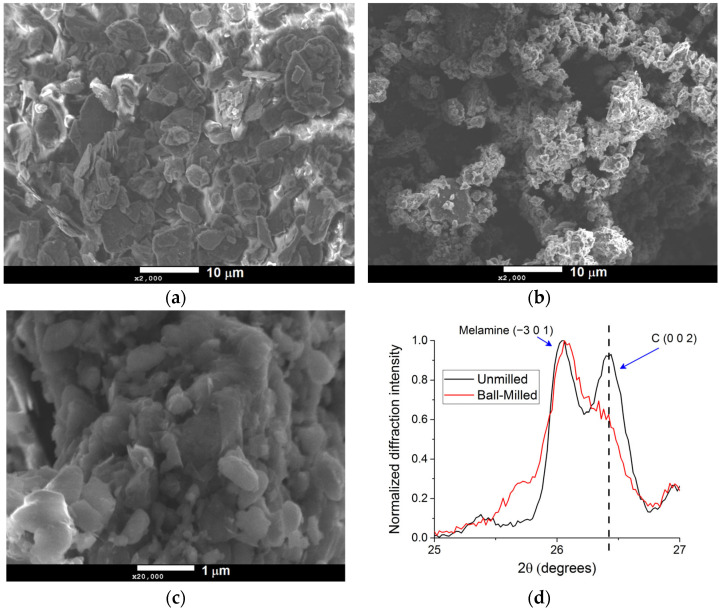
(**a**) SEM image of unmilled graphite/melamine, (**b**) SEM image of milled Graphite/melamine sample, (**c**) SEM image of milled Graphite/melamine sample at a higher magnification and (**d**) Comparison of the XRD patterns of unmilled (black line) and milled (red line) samples. The dash line indicates the position of the C (002) pattern.

Figure 1 presents the top-view SEM images of graphite/melamine powder before (Figure 1a) and after 1-hour ball milling (Figure 1b) at the same magnification (×2000). Figure 1c gives the top view of the milled sample at a higher magnification (×20,000). The SEM images show that the grain size is reduced significantly to less than 1 µm though high-energy milling, despite the relatively short milling duration. In fact, similar results were reported Xue et al. for 48 hours milling times when using planetary ball mills (low energy), despite using a ball-to-powder ratio that is larger than the one we used in the present study [11]. The rapid reduction in the grain size through ball milling indicates that the shaker mill was operating in the high-energy mode of milling dominated by the fracture mode. Additionally, the presence of sub-micron graphene sheets could be confirmed in Figure 1c, even though there was a high degree of agglomeration typical of the as-milled powders morphology.

The effect of the high-energy ball milling was investigated using X Ray Diffraction. Figure 1d shows the comparison of the diffractograms of the pristine graphite/melamine powders and the milled sample. The diffractograms were normalized to the maximum. As can be seen on the diffractograms, Melamine (C_3_H_6_N) main peak (−301) located at 2θ ≈ 26.1° was identified using the ICDD#00-024-1654 reference patterns, while Graphite main peak (002) located at 2θ ≈ 26.4° was identified using the ICDD#00-001-0640 reference patterns. The (002) peak of graphite broadens, and its intensity decreases after milling. This is indicative of the exfoliation of the graphene sheets through high-energy milling in presence of melamine since the broadening of the (002) peak of graphite is related to the out-of-plane crystallite size that is proportional to number of the graphene sheets stacked in graphite. A broadening if the (−301) peak is melamine is observed, indicating a decrease in the crystallite size of melamine via high-energy ball milling.

**Figure 2:**
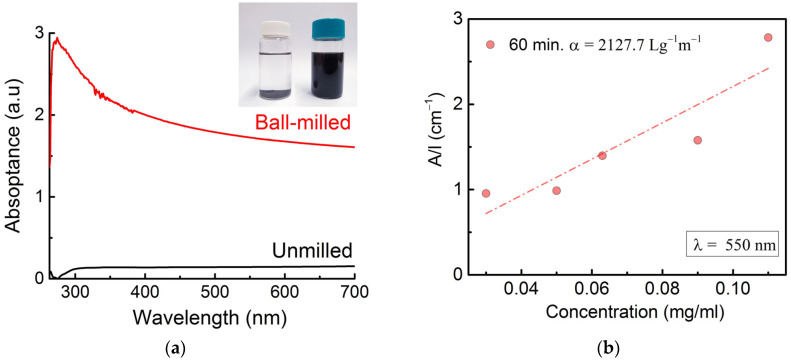
(**a**) Comparison of the UV-Vis-NIR spectra of unmilled and milled sample suspensions in DMF (The inset in Figure 2a Photographs of dispersions of unmilled (left vial) and milled powders (right vial) in DMF), and (**b**) optical absorbance at λ = 550 nm divided by cell length (A/l) versus concentration of the ball-milled sample.

An amount of 7 mg of unmilled or ball-milled powders were sonicated in 20 ml of DMF to produce suspensions. No dispersion was observed for the unmilled powders (inset in Figure 2a, left vial), and a black suspension is obtained for the ball-milled powders (inset in Figure 2a, right vial). The latter is indicative of the formation of graphene. It is worth mentioning that the suspension made of ball-milled powders has been stable since its preparation several months ago.

The suspensions were also characterized by UV-Vis-NIR spectrophotometry. The corresponding spectra are given on Figure 2a. While no absorption is obtained for the suspension of the unmilled powder, the suspension of the ball-milled powder shows a single peak around 274 nm, characteristic of π-π* peak of multilayer graphene [18]. Figure 2b indicates the Beer–Lambert behavior of the ball milled powders at 550 nm, where the optical absorbance divided by the cell length is plotted as a function of the graphene concentration.

**Figure 3:**
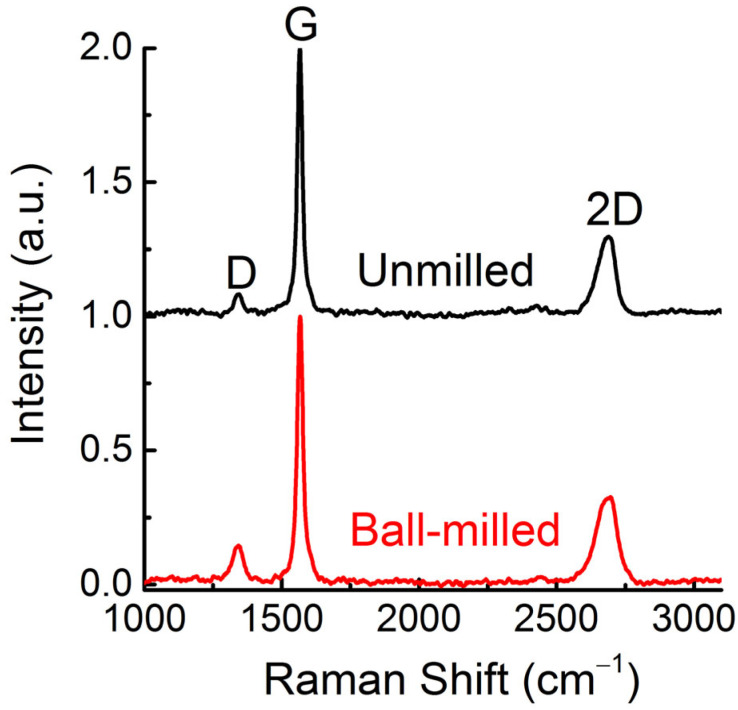
Comparison of the Raman spectra at 532 nm of unmilled and milled sample (after washing).

Figure 3 compares the 532 nm Raman spectra of the pristine graphite and the milled powders after washing with hot water to remove melamine. The most intense peak around 1570 cm^-1^ is the G peak, and it is due to the bond stretching of all pairs of sp^2^ atoms in both rings and chains [19,20]. The peak observed at about 1340 cm^−1^ is D peak characteristic of the breathing mode of sp^2^ rings. This mode is defects-activated due to impurities, edges, and finite size effects [17,19,20]. The 2D peak is the D-peak overtone and it is always present as there is no need for defects to activate it, which explains why it is always present [20]. The integrated intensity ratio I_D_/I_G_ increases from 0.087 for the pristine graphite to 0.262 for the milled sample, indicating a decrease in the in-plane average size of the breathing domains. In fact, the average in-plane size $L_{a}$ estimated using Equation (1) is 221 nm for the pristine graphite and 73 nm for the milled sample. This result signifies the creation of in-plane defects in the graphene sheets through the milling process.

**Figure 4:**
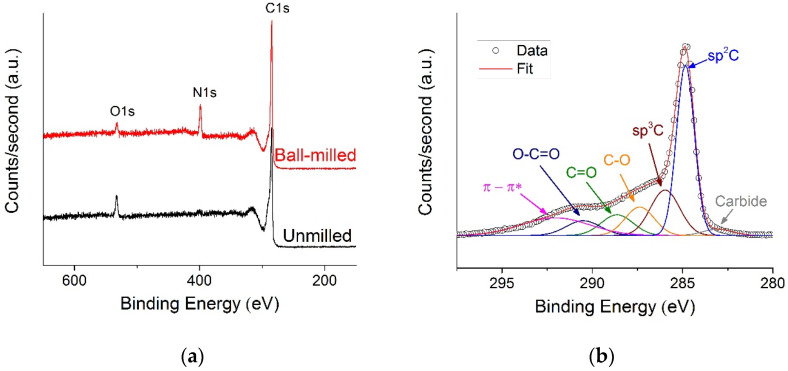

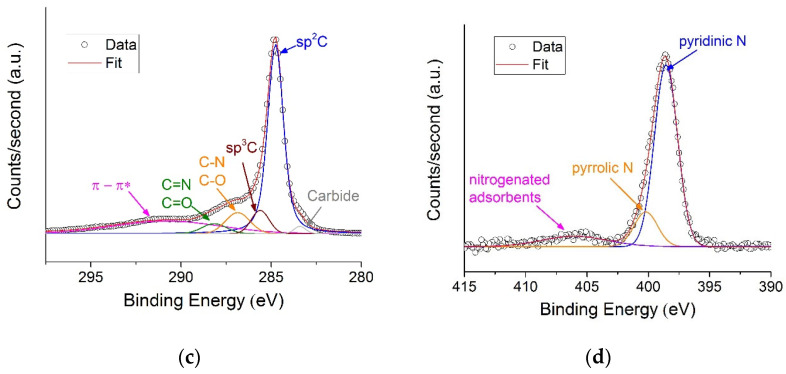
(**a**) Comparison of XPS survey spectra of unmilled and ball-milled powders (after washing with water) (**b**) deconvolution of C1s peak of unmilled sample (**c**) deconvolution of C1s peak of the ball-milled powder and (**d**) deconvolution of N1s peak of the ball-milled powder.

The results of the X-ray photoelectron spectroscopy of the unmilled and ball-milled powders after washing with hot water are shown in Figure 4. The survey spectra (cf. Figure 4a) of the surface composition (i.e., first few nanometers as per the XPS sensitivity) of pristine (i.e., unmilled) graphite indicates the presence of C1s and O1s peaks. XPS survey spectra of the milled sample also show the presence of C1s and O1s peaks. N1s peak is clearly observable on the milled powder XPS spectrum around 400 eV. As for the unmilled graphite, the XPS survey spectrum seems to display a minor feature around 400 eV, probably due to the presence of a negligible amount of melamine adsorbed on graphite grains during the washing process. The atomic content of the samples calculated from the XPS survey spectra is given on Table 1. The contents in both C and O on the surface of pristine graphite decrease in favor of the content in N after high-energy ball milling of graphite in presence of melamine.

The deconvolution of the C1s spectral line of the unmilled graphite shown on Figure 4b reveals the presence of peaks around 284.8, 285.9, 287.4, 288.6 and 290.5 eV that could be attributed to sp^2^ C, sp^3^ C, C–O C=O and O–C=O, respectively [21,22]. The broad peak centered around 292.0 eV is attributed to the π to π* transition [22]. Another small intensity peak observed around 283.1 eV is attributed to the existence of a carbide phase [23]. The deconvolution of the C1s spectral line of the ball-milled powder shown in Figure 4c shows the presence of peaks at 284.8, 285.6, 286.9 and 288.2 eV that could be attributed to sp^2^ C, sp^3^ C, C–N/C–O and C=N/C=O, respectively. The broad peak corresponding to π to π* transition is still observed around 291.0 eV, and the carbide peak is also observed at 283.4eV. No O–C=O peak is observed after the deconvolution of the C1s spectral line of the milled sample. 

Remarkably, the ratio of the content in sp^2^ C, corresponding to the in-plane graphene bonding [24], to that in sp^3^ C, corresponding to the out-of-plane bonding between the graphene sheets [24], increases from 2.29 before milling to 8.81 after milling. This result is indicative of the exfoliation of graphite into graphene. The carbide content is primarily due to the existence of metallic impurities that were initially present in the pristine graphite/melamine mixture (mainly Fe) as revealed by Inductively Coupled Plasma-optical emission spectroscopy (ICP-OES) measurements (cf. Supplementary Materials, Table S1). 

The deconvolution of the N1s spectral line for the ball-milled powders reveals the existent of three peaks (cf. Figure 4d). The first peak at 398.6 eV corresponds to pyridinic configuration of N atoms. The second peak at 400.2 eV is indicative of pyrrolic configuration of N atoms [11,25]. The third peak, displaying as a broad feature around 406 eV, is related to the existence of nitrogenated adsorbents [25]. From these results, it appears that N-doping of graphene through high-energy ball milling of graphite in presence of melamine is mainly a pyridinic doping. Similar results regarding N-doping of graphene were reported in the literature by Xue et al. for graphite milled in presence of melamine at the ratio graphite/melamine of 1:10 for a duration of 48 hours in a planetary ball mill [11]. In our case, milling for only 1 hour with a graphite/melamine ratio of 1:3 was enough, demonstrating the efficiency of the high-energy ball milling in a shaker mill as compared to planetary ball mill. In addition, our results show a nitrogen content in the ball milled sample of 12.95%, which is slightly higher than the one reported by Xue et al. (11.4%). Finally, Xue et al. reported the existence of graphitic nitrogen as one of the dopant species, while in our case, no graphitic nitrogen was revealed by XPS (cf. Figure 4d).

**Figure 5:**
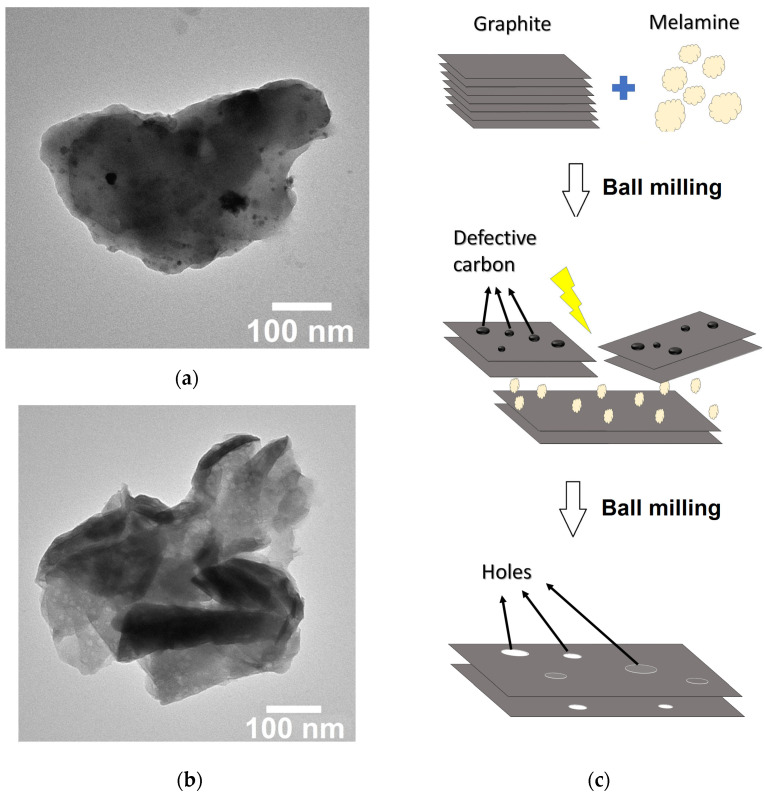
(**a**) TEM image of defective multilayer graphene as an intermediate product, (**b**) TEM image of the holey multilayer graphene, and (**c**) schematic of the synthesis process of holey multilayer graphene via high-energy ball milling in presence of melamine.

To have more insights regarding the nano-morphology of the ball-milled sample, TEM images were performed after the washing step. The results show the presence of two types of multilayer graphene. The first type shown in Figure 5a corresponds to the so-called defective graphene because of the presence of defective carbon in the basal plane of the multilayer graphene sheets. The second type is the multilayer holey graphene characterized by in-plane perforation of the multilayer graphene sheets (cf. Figure 5b). It is known from the literature that one of the routes for the preparation of holey graphene is the removal of the defective carbon by annealing defective graphene at an elevated temperature [4]. In the present work, the removal of the defective carbon regions could be achieved by high-energy ball milling. 

Based on the characterization results described in this work, a process scheme of the formation of N-doped multilayer graphene is proposed in Figure 5c. Starting from a mixture graphite/melamine (cf. Figure 5c), high-energy ball milling induces the fragmentation of graphite. This fragmentation is controlled because of the presence of melamine that acts as a diluent to avoid the production of amorphous carbon. Simultaneously, an exfoliation of graphene sheets takes place through the adsorption of melamine onto the graphene sheets to break the van der Waals forces holding the sheets together in the graphitic structure. The repeated shocks during the high-energy ball milling process favors the production of defective multilayer graphene sheets because of the out-of-equilibrium nature of the process. As the ball milling process goes on, the defects present in the basal plane of the defective graphite are the ones likely to be removed via ball milling because of their poor mechanical properties as compared to graphene. Moreover, since the ball milling is performed in air and in presence of melamine, the removal of the defective regions could be facilitated by the presence of oxygen and/or by the nitrogen because defects tend to be highly reactive. As a result, multilayer holey graphene is produced. Because the N-doping was found to be mainly pyridinic, with the existence of some pyrrolic species, we could conclude that N atoms are mainly distributed around the pores and the edges of the multilayer holey graphene. The presence of pores could also explain the high content in nitrogen in the ball-milled sample. 

## 4. Conclusions

We have developed a new method for the preparation of N-doped multilayer holey graphene using shaker-type high-energy ball milling of graphite as the starting material in presence of melamine. A content in nitrogen as high as 12.96 at.% was achieved. A step-by-step process scheme was also proposed and discussed at the light of the different characterizations.

While the methods reported in the literature for the preparation of holey graphene suffer from drawbacks limiting their effective implementation in industry such as the high cost of the precursors, the complex steps, the annealing at high temperatures and, very often, the involvement of hazardous chemicals and/or byproducts, the method that we developed is based on an all-solid, one-step milling process of graphite in the presence of melamine and taking place at ambient conditions (air, atmospheric pressure, room temperature) for 1 hour, making it simple and cost effective. Moreover, it is eco-friendly as it does not involve the use of hazardous chemicals or the generation of harmful byproducts. The proposed method is also versatile because the structure of the multilayer holey graphene, its yield and the nitrogen doping content could be further optimized through the control of parameters such as graphite/melamine weight ratio, balls-to-powder weight ratio, the milling balls and/or milling vial hardness, size of the balls and/or their size distribution in the vial and the milling time. Finally, the proposed method is scalable.

## Figures and Tables

**Table 1:** Atomic content from XPS survey spectra.

Sample	C (%)	O (%)	N (%)
Pristine (unmilled) graphite	92.57	7.43	-
Ball milled sample	84.95	2.09	12.96

## Data Availability

Not applicable.

## Supplementary Materials

The following supporting information can be downloaded at: https://www.mdpi.com/article/10.3390/ma16010219/s1, Table S1: ICP-OES analysis results of the pristine mixture and the ball-milled sample.
