# Supplementary material for: Preparation of Nitrogen-doped Holey Multilayer Graphene Using High-Energy Ball Milling of Graphite in Presence of Melamine
Source: Materials (Basel). 2022 Dec 26;16(1):219. doi: 10.3390/ma16010219 (PMC9822149; doi:10.3390/ma16010219)
Supplement: Supplementary file 1 [file materials-16-00219-s001.zip › materials-2113450-Supplementary_Material.pdf]

Preparation of nitrogen-doped holey multilayer graphene using high-energy ball milling of graphite in presence of melamine

(supplementary material)

Ali Hendaoui [1,*] and Abdullah Alshammari [2]

Inductively coupled plasma - optical emission spectroscopy (ICP-OES) analysis:

Pristine (unmilled) mixture and ball milled sample were analyzed using a PerkinElmer OPTIMA 2000™ ICP optical emission spectrometer (PerkinElmer Inc., Waltham, MA, USA) to identify the impurities content. The corresponding results are listed in Table S1.

Table S1. ICP-OES analysis results of the pristine mixture and the ball-milled sample.

**Analyte**	**Concentration in pristine (unmilled) mixture µg/L**	**Concentration in the ball-milled sample µg/L**
Barium (Ba)	5.5	5.2
Iron (Fe)	2107	2194
Cadmium (Cd)	5	5
Zinc (Zn)	503	562
Manganese (Mn)	37.5	36.3
Beryllium (Be)	0	0
Aluminum (Al)	1056	1103
Cobalt (Co)	1.9	3
Lead (Pb)	93	76
Arsenic (As)	0	0
Molybdenum (Mo)	21	27
Selenium (Se)	0	0
Antimony (Sb)	31.2	28
Nickel (Ni)	13.6	19
Vanadium (V)	7.7	7
Copper (Cu)	34.7	36.7
